# Increasing Hospital Admissions for Pneumonia, England

**DOI:** 10.3201/eid1405.071011

**Published:** 2008-05

**Authors:** Caroline L. Trotter, James M. Stuart, Robert George, Elizabeth Miller

**Affiliations:** * University of Bristol, Bristol, UK; † Health Protection Agency, Stonehouse, Gloucestershire, UK; ‡ Health Protection Agency Centre for Infections, London, UK

**Keywords:** Pneumonia, hospital, patient admission, epidemiology, trends, England, research

## Abstract

Pneumonia is an important cause of illness and death in England. To describe trends in pneumonia hospitalizations, we extracted information on all episodes of pneumonia that occurred from April 1997 through March 2005 recorded in the Hospital Episode Statistics (HES) database by searching for International Classification of Diseases 10th revision codes J12–J18 in any diagnostic field. The age-standardized incidence of hospitalization with a primary diagnosis of pneumonia increased by 34% from 1.48 to 1.98 per 1,000 population between 1997–98 and 2004–05. The increase was more marked in older adults, in whom the mortality rate was also highest. The proportion of patients with recorded coexisting conditions (defined by using the Charlson Comorbidity Index score) increased over the study period. The rise in pneumonia hospital admissions was not fully explained by demographic change or increasing coexisting conditions. It may be attributable to other population factors, changes in HES coding, changes to health service organization, other biologic phenomenon, or a combination of these effects.

## Abstract 2

This rise in recorded incidence from 2001 to 2005 was particularly marked among the elderly.

Community-acquired pneumonia is an important cause of illness and death in the United Kingdom, particularly for elderly adults. In recent years, increases in hospital admissions for pneumonia have been noted in the United States (*1*,*2*), Denmark (*3*), and the Netherlands (*4*). It has been suggested that this rise is due to an aging population and an increased prevalence of coexisting conditions (such as diabetes and chronic obstructive pulmonary disease); however, these factors seem to only partially explain the observed increase in pneumonia hospitalizations (*2*). Furthermore, these increases have occurred despite widespread influenza and pneumococcal vaccination programs that target the elderly. *Streptococcus pneumoniae* is a leading cause of community-acquired pneumonia (*5*,*6*), but the 23-valent pneumococcal polysaccharide vaccine currently recommended for the elderly in the United Kingdom has little efficacy against nonbacteremic pneumonia (*7*). A 7-valent pneumococcal conjugate vaccine (PCV7) was introduced into the United Kingdom infant immunization schedule in September 2006 (*8*). Experience with PCV7 in the United States suggests that, by reducing carriage and thus the opportunity for transmission of vaccine types, vaccination can lead to a reduction in invasive pneumococcal disease (*9*) and pneumonia (*10*) in unvaccinated cohorts. We report on the epidemiology of pneumonia before PCV7 vaccination was introduced by examining trends in hospital admissions for pneumonia in England during an 8-year period (April 1997 through March 2005).

## Materials and Methods

The Hospital Episode Statistics (HES) from the National Health Service (NHS) Information Centre for Health and Social Care contains details of all admissions to NHS hospitals in England (www.hesonline.org.uk). The database holds information on patient characteristics and clinical diagnoses and procedures, in addition to geographic and administrative data. Diagnoses in HES are recorded in up to 14 diagnostic fields by using the International Classification of Diseases, 10th revision (ICD-10), coding system.

Each record in the HES database relates to 1 “finished consultant episode.” This is the period a person spends under the care of 1 NHS consultant during a single hospital admission; multiple episodes may occur within 1 admission. We identified all episodes of pneumonia from April 1997 through March 2005 by searching the HES database for ICD-10 codes J12–J18 in any of the 14 diagnostic fields. Episodes were classified into those with pneumonia as a primary diagnosis and those with pneumonia listed in any diagnostic field. A patient identifier—based on date of birth, postal code, and sex—was created for each episode and encrypted to ensure anonymity. This patient identifier was then used to identify and order the number of episodes for each patient within 1 HES year, which runs from April through March. For our analyses, we retained only the first episode for each patient because the main purpose of this analysis was to identify the number of persons admitted with pneumonia at least once a year, rather than to identify multiple episodes or admissions for the same patient. Because deaths may occur in episodes subsequent to the first, we identified patients who died in hospital with an ICD-10 code for pneumonia in any diagnostic field within 30 days of the first admission with pneumonia. (Note that in-hospital deaths that were not associated with pneumonia and deaths that occurred outside of hospital were not identified.)

To adjust for coexisting conditions, we computed the Charlson Comorbidity Index score for each patient and grouped this into 4 levels: no coexisting conditions and mild, moderate, and severe coexisting conditions (*11*,*12*). This index includes 19 major disease categories and has been adapted and validated for use with hospital discharge data in ICD databases (*13*). Excess alcohol consumption is also a risk factor for pneumonia, but the Charlson index does not specifically include codes for alcohol use or alcohol-related illness. To address this, we also searched for the presence of >1 of the following alcohol-related ICD-10 codes within each episode: F10*, G31.2, G62.1, I42.6, K29.2, K70*, K860, T51*, X45*, X65*, Y15*, Y919, Z721 (* indicates that either a 3-digit code is valid or that all 4th digits are valid).

Length of stay is recorded in HES and is equal to the difference between admission date and discharge date (where both are recorded). Differences between the median length of stay by year and age group were assessed by using a nonparametric equality-of-medians test.

Mid-year population estimates for England for 1997 to 2004, stratified by 5-year age groups and sex, were obtained from the Office for National Statistics (ONS). The annual incidence rates of hospitalization for pneumonia were calculated overall and stratified by age by using ONS population statistics as a denominator. Age-standardized incidence rates were calculated by using the 1997 English population as the reference standard. To identify seasonal patterns, the weekly number of admissions was summarized, and a graph was created that showed the 4-week moving average number of pneumonia admissions (stratified by patients <65 years and >65 years). We used multivariable logistic regression to examine changes in the odds of death (within 30 days after hospital admission for pneumonia) over time (years), controlling for known risk factors, e.g., age group, sex, and coexisting conditions. All analyses were performed by using Stata version 9.2 (StataCorp LP, College Station, TX, USA).

## Results

### Incidence of Pneumonia (Primary Diagnosis)

**Figure 1:**
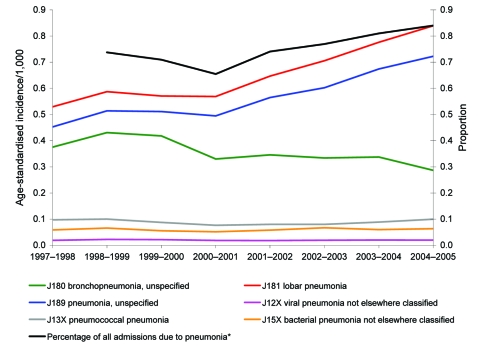
Trends in age-standardized incidence of hospital admission with a primary diagnosis of pneumonia-specific International Classification of Diseases (10th revision) codes, by Hospital Episode Statistics year (April to March). *Additional data on percentage of all admissions due to pneumonia published by the Information Centre for Health and Social Care (www.ic.nhs.uk). Data not available for 1997–98.

The number of patients admitted to an NHS hospital in England at least once per year with a primary diagnosis of pneumonia increased from ≈72,060 in 1997–98 to 101,381 in 2004–05. Overall, the age-standardized incidence rate rose by 34% over the study period from 1.48 per 1,000 population to 1.98 per 1,000 population (Table 1). This increase was noted in all age groups but was most marked in older adults. The age-specific incidence of hospitalization with pneumonia as a primary diagnosis was 7% higher overall for male patients than for female patients over the study period. The percentage of total admissions that were due to pneumonia increased over the study period (Appendix Figure 1). Figure 1 shows the trends in specific diagnoses of pneumonia. Most of the increase over the study period was observed after 2000–2001 in just 2 codes, J181 (lobar pneumonia, unspecified) and J189 (pneumonia, unspecified). Diagnoses of J180 (bronchopneumonia, unspecified) decreased over the study period. Only 6% of episodes with pneumonia as a primary diagnosis had a causative pathogen within that primary diagnostic code. Of these, the most common organisms specified were *S. pneumoniae* (37%), *Mycoplasma pneumoniae* (26%), Streptococci other than group B and *S. pneumoniae* (10%), *Staphylococcus* spp. (8%), and *Haemophilus influenzae* (8%).

**Table 1:** Age-standardized incidence of hospital admission for pneumonia (primary diagnosis) by age group per 1,000 population, England*

HES year (April through March)	All ages	<65 y	65–74 y	75–84 y	>85 y
1997–98	1.48	0.70	2.63	6.84	15.99
1998–99	1.67	0.72	3.02	8.08	18.59
1999–2000	1.62	0.65	3.04	8.05	18.80
2000–01	1.50	0.65	2.67	7.08	16.75
2001–02	1.67	0.71	3.02	7.73	18.89
2002–03	1.77	0.75	3.20	8.14	19.62
2003–04	1.91	0.78	3.46	8.79	22.41
2004–05	1.98	0.84	3.55	8.77	22.18
% change from 1997–98 to 2004–05	34	20	35	28	39

*HES, Hospital Episode Statistics.

### Pneumonia in Any Diagnosis

The number of hospitalizations for which pneumonia was listed in any diagnostic field increased from 110,143 to 153,312; the equivalent age-standardized rate increased 32%, from 2.26 per 1,000 to 2.98 per 1,000 population over the same period. The trends in the rate of hospitalization with at least 1 pneumonia episode per year were very similar when we compared pneumonia as a primary diagnosis with any listed diagnosis (data not shown).

### Coexisting Conditions

**Figure 2:**
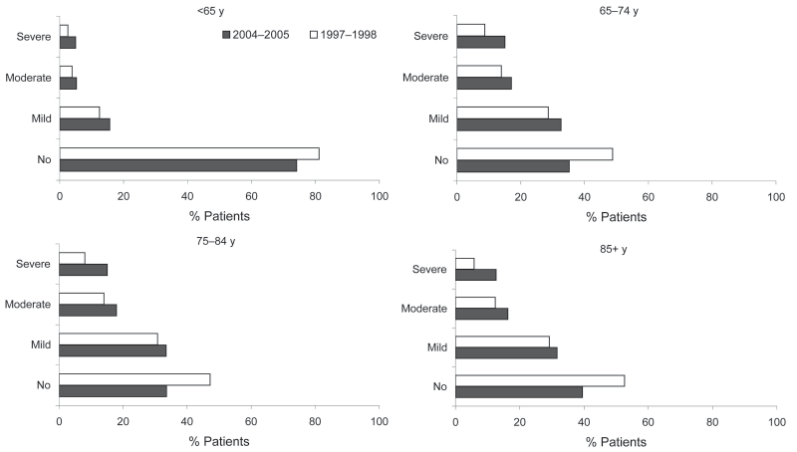
Percentage of patients admitted to hospital with a primary diagnosis of pneumonia with coexisting conditions, as defined by using the Charlson Comorbidity Index, by age group.

The proportion of patients with a primary diagnosis of pneumonia and coexisting conditions (defined by Charlson Comorbidity Index score) varied over time and by age (Figure 2). In all age groups, the proportion of patients with coexisting conditions increased between 1997–98 and 2004–05. The median number of ICD10 diagnoses recorded increased from 2 in 1997–98 to 3 in 2004–05 in patients with a primary diagnosis of pneumonia, and from 3 to 4 in those with pneumonia in any diagnostic field. In each year, <1% of patients overall (range 0.5%–1%) had an additional alcohol-related code recorded. These codes were slightly more common (range 0.9%–1.9%) in those <65 years of age compared to those >65 years of age (range 0.3%–0.5%).

### Length of Stay

Length of stay was recorded in HES for 73% of admissions. Where reported, the median duration of stay (all ages) for those with a primary diagnosis of pneumonia was 5 days for all years apart from 1999–2000 (6 days, p<0.001 compared to 1997–98) and 2004–05 (4 days, p<0.001 compared to 1997–98). For all-cause admissions, the median length of stay was 2 days each year in the study period. The median duration of stay in hospital increased with age. For those with a primary diagnosis of pneumonia, length of stay was 3 days for patients <65 years of age, 6 days for those 65–74 years, 8 days for those 75–84 years, and 9 days for those >85 years (p<0.001).

### Admission Patterns

To assess any possible changes in admission practices and patterns, we analyzed the proportion of weekend admissions compared to those at midweek. Overall, the proportion of admissions with a primary diagnosis of pneumonia that occurred on a Saturday or Sunday only changed from 23% to 24% between 1997–98 and 2004–05. In adults >85 years of age, the increase in weekend admissions was slightly greater, increasing from 22.5% in 1997–98 to 25.2% in 2004–05 (p<0.001).

A clear seasonal variation in admissions for pneumonia occurred; incidence was highest in the winter months. Seasonal patterns were similar for those <65 of age and those >65 years of age (Appendix Figure 2).

### Mortality Rates

**Figure 3:**
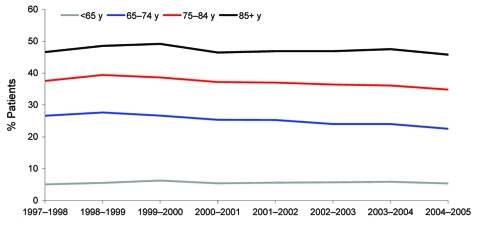
Percentage of patients admitted with a primary diagnosis of pneumonia who died in hospital with pneumonia within 30 days of their first pneumonia admission, by Hospital Episode Statistics year (April to March).

The crude 30-day in-hospital mortality rates for all ages were fairly stable over the study period, declining slightly in older adults (Figure 3). Mortality rates were higher in the older age groups; in adults >85 years of age; 47% were reported to have died in the hospital within 30 days of admission with a primary diagnosis of pneumonia over the study period.

After age, sex, and Charlson Comorbidity Index score were controlled for, the odds of death within 30 days were significantly higher in 1998–99 and 1999–2000 compared to 1997–98, and significantly lower from 2000–01 onwards (Table 2). The odds of death were lowest in 2004–05, although this finding may partly reflect censoring of the data at the end of the study period. The odds of death rose with severity of the Charlson Comorbidity Index score and with increasing age (Table 2).

**Table 2:** Odds of death within 30 days of admission for hospitalized patients with a primary diagnosis of pneumonia*

Variable	No. deaths (%)	Adjusted odds ratio† (95% CI)
Year (April to March)		
1997–98	17,743 (24.8)	1.00
1998–99	22,152 (27.2)	1.07 (1.05–1.10)
1999–2000	22,425 (28.2)	1.07 (1.04–1.10)
2000–01	19,392 (26.1)	0.94 (0.92–0.97)
2001–02	22,198 (26.5)	0.93 (0.90–0.95)
2002–03	23,352 (26.2)	0.89 (0.87–0.91)
2003–04	25,946 (26.7)	0.88 (0.86–0.90)
2004–05	25,379 (25.0)	0.80 (0.78–0.82)
Age, y		
<65	13,576 (5.6)	1.00
65–74	25,349 (25.1)	4.34 (4.25–4.44)
75–84	67,526 (37.0)	7.77 (7.61–7.93)
>85	72,136 (47.2)	12.81 (12.55–13.08)
Sex		
M	87,132 (25.2)	1.00
F	91,455 (27.6)	0.97 (0.96–0.98)
Comorbidity index		
None	63,254 (16.9)	1.00
Mild	54,030 (31.9)	1.59 (1.56–1.61)
Moderate	33,598 (42.9)	2.47 (2.42–2.51)
Severe	27,705 (50.1)	3.72 (3.65–3.80)

*Results from a multivariable logistic regression. CI, confidence interval. †Model includes year, age, sex, and coexisting conditions.

## Discussion

The age-standardized incidence of hospital admissions for pneumonia in England increased 34% between 1997–98 and 2004–05. The increase occurred particularly in nonspecific codes—lobar pneumonia, unspecified; and pneumonia, unspecified—and was more marked among older age groups. The proportion of patients with recorded coexisting conditions increased over the study period; alcohol-related codes were recorded infrequently. In-hospital deaths among patients admitted with pneumonia were high, particularly in the most elderly and those with severe coexisting conditions. After adjustment, the odds of death were lower in more recent years compared to 1997–98.

The magnitude of the increase in hospital admission is similar to that reported in the United States (*1*) and Denmark (*3*). Our findings also correspond with the reported rise in the extent of pneumonia in adult intensive care units in the United Kingdom, where community-acquired pneumonia admissions rose by 128% between 1996 and 2004, compared to a rise in total admissions of 24% (*14*). In contrast to hospital admissions, reports from primary care show that consultations for respiratory tract infections as a whole have markedly decreased (*15*,*16*) and that the overall mean weekly incidence of “pneumonia and pneumonitis” decreased from 3.69 per 100,000 in 1997 to 1.39 per 100,000 in 2005 (data from the Birmingham Research Unit of the Royal College of General Practitioners, www.rcgp.org.uk/bru_/bru_home.aspx). In adults >75 years of age, the mean weekly incidence declined from 16.9 and 14.6 per 100,000 (in men and women, respectively) in 1999 to 6.5 and 5.5 per 100,000 in 2005.

Whether the decline in the case-fatality rate over the study period is due to improved care leading to better outcomes, or to patients with less severe pneumonia being admitted, is not clear. The number of ONS-registered deaths with pneumonia as an underlying cause in England has fallen since 2001, but this is thought to be primarily due to changes in the rules for classifying deaths and the switch from ICD-9 to ICD-10 coding in 2001 (*17*). A reduction in the length of stay in hospital may also indicate that less severe case-patients are being admitted to hospital, but we found only slight variation in the median length of stay over time (i.e., 6 days vs. 4 days).

The rise in pneumonia hospitalizations may be attributable to population factors, changes in HES coding, changes to health service organization, other biologic phenomenon, or a combination of these effects. We assessed some of these factors with our database, and we discuss possible explanations for the observed trends in pneumonia below.

Age, coexisting conditions, and residence in nursing homes are among the established risk factors for community-acquired pneumonia (*18*). The United Kingdom has an aging population; ONS population statistics for 1997 through 2004 show a 13% increase in the number of adults in England >80 years of age and a 25% increase in those >90 years. To control for this fact, we present our results as age-standardized incidence, although we cannot take into account other secular trends, such as increasing numbers of elderly persons living alone with little support. The proportion of patients with reported coexisting conditions increased over the study period in all age groups. This may reflect a true increase in the prevalence of heart disease, diabetes, and other conditions in the population (*19*), but it may also be a reflection of improved coding in HES, as the median number of diagnoses per patient increased over the study period. Because the residential status of patients is not recorded in HES, we could not analyze the impact of place of residence directly, but there has been little recent change in the numbers and proportions of elderly persons living in residential care (*20*).

HES is essentially an administrative database; nevertheless, it has been widely used for epidemiologic studies. Diagnoses are recorded by coding clerks who review patient case notes, rather than by the attending physician, and some variability in the quality and consistency of coding is likely. We attempted to control for this variability by principally reporting on case-patients with pneumonia as a primary diagnosis. We also demonstrated that the proportion of admissions attributed to pneumonia increased over the period. Nevertheless, we have not attempted to verify the accuracy of the diagnoses recorded in HES. Since our analyses are based on the first admission for pneumonia in a year, most cases are likely to be community-acquired, but we have not excluded some potential sources of hospital-acquired pneumonia, e.g., patients recently hospitalized for reasons other than pneumonia before their pneumonia episode.

Changes in healthcare organization in England may have contributed to some of the increase in admissions and may help to explain the contrasting trends in hospital admission and primary care consultations. General practitioners are no longer obliged to provide after-hours care, so patients with pneumonia may go directly to hospital, or be seen by an unfamiliar doctor who may be more likely than the patient’s usual general practitioner to admit him or her to hospital. The proportion of admissions occurring on a weekend (a proxy for after-hours admissions because HES only contains information on the date of admission and not the time) changed little overall, and only increased slightly, from 22.5% to 25.2%, between 1997–98 and 2004–05 for adults >85 years. The high in-hospital death rate observed in this age group may also reflect people’s unwillingness to have a patient die at home; data on place of death suggest that the percentage of deaths (from all causes) in England and Wales that occurs outside a hospital or other medical establishment fell slightly, from 30.6% in 1998 to 25.9% in 2004 (*21*). The level of care available to elderly patients has also improved, as evidenced by the introduction of the National Service Framework for older people in 2001 and an increasing number of specialists in geriatric medicine (*22*). Expectations of patients and families may also be changing. Organizational changes may be contributing to the observed trends; however, similar trends have been observed in other countries, so this is unlikely to be the sole explanation.

A range of organisms are implicated in the etiology of community-acquired pneumonia. These include *S*. *pneumoniae*, *M. pneumoniae*, *H*. *influenzae*, *Chlamydia* species, *Legionella* species, *Staphylococcus aureus,* and respiratory viruses (influenza, respiratory syncytial virus [RSV], adenovirus, parainfluenza) (*6*,*23*,*24*). In this study, only 6% of hospital admissions had a specific pathogen identified in the primary diagnostic code. The absence of microbiologic data in these cases means that indirect methods must be used to investigate the underlying etiology. For example, Muller-Pebody et al. (*25*) used seasonal regression models to estimate that 42% of hospital admissions for unspecified pneumonia were attributable to *S. pneumoniae*, 10% to influenza, 9% to *H. influenzae*, 7% to *Bordetella pertussis,* and 5% to RSV. Further analysis is required to investigate whether such model estimates are similar when more recent HES data are used.

Another factor to consider is that community prescribing of antimicrobial agents has decreased substantially in the United Kingdom in recent years (*26*). Reduced usage of antimicrobial agents in general practice may be related to increased pneumonia deaths (*27*). The cause of this reduction in usage of antimicrobial agents, either as a result of higher prescribing thresholds or fewer consultations for respiratory illness, has been debated (*16*,*27*–*29*). In either case, a plausible argument can be made that reduced use of these agents may result in increased or prolonged (asymptomatic) carriage and thus transmission of common respiratory pathogens that cause pneumonia in the population. This ecologic effect is difficult to substantiate, and reductions in antimicrobial agents seem to predate the rise in pneumonia hospitalizations. Nonetheless, international comparisons of pneumonia hospitalizations and drug-prescribing trends may be enlightening.

The increase in pneumonia admissions has occurred despite increasing coverage for influenza and pneumococcal vaccinations in the elderly (*30*,*31*). Since 1992, a 23-valent pneumococcal polysaccharide vaccine (PPV23) has been recommended in England for persons at high risk, and in 2003, this recommendation was extended to include all persons >65 years of age. This universal program was phased in over 3 years, targeting those >80 years of age from August 2003, >75 years of age from April 2004, and >65 years of age from April 2005. Although PPV23 offers a modest level of protection against invasive disease, the vaccine appears to have little benefit against pneumonia (*7*). A person’s vaccination status is not recorded in HES so we could only explore the association between PPV23 and pneumonia at an ecologic level. We did not observe any associations between the trends of PPV23 coverage and overall pneumonia incidence (ICD-10 codes J12–J18). PPV23 coverage increased steadily from 1995 onwards (*30*); from 2003 onwards, coverage increased in the age groups targeted in the universal program. By contrast, the incidence of pneumonia increased in all age groups from 2001–02 onwards. The trend in pneumococcal pneumonia (ICD-10 code J13X), which is most likely to represent confirmed pneumococcal pneumonia, was stable, suggesting that PPV23 had little influence. The experience of the United States leads us to expect that the introduction of the 7-valent pneumococcal conjugate vaccine (PCV7) into the United Kingdom infant immunization schedule in September 2006 will result in a reduction in the transmission of pneumococcal vaccine serotypes and subsequent reductions in invasive (*32*–*34*), and to a lesser extent noninvasive, pneumococcal disease (including pneumonia) (*10*) even in unvaccinated persons. Analysis of temporal trends in *Pneumococcus*-attributable illnesses pre- and post-PCV7 introduction will help to quantify these effects in England.

## Conclusion

We have observed a 34% increase in the incidence of hospital admissions for pneumonia in England in recent years, particularly in older adults. We believe this increase is real; however, the trends are not fully explained by an aging population, rising prevalence of coexisting conditions, or coding changes. Further research is required to understand the reasons for the increase in pneumonia hospitalizations so that the most appropriate interventions can be determined.

## Supplementary Material

Appendix Figure 1 Percentage of total hospital admissions that were due to pneumonia between 1998–99 and 2004–05, by age group.

Appendix Figure 2 Seasonal patterns of hospital admissions with a primary diagnosis of pneumonia, by age group (4-week moving average).
