# Supplementary figures and images for: Increasing Hospital Admissions for Pneumonia, England
Source: Emerg Infect Dis. 2008 May;14(5):727–33. doi: 10.3201/eid1405.071011 (PMC2600241; doi:10.3201/eid1405.071011)

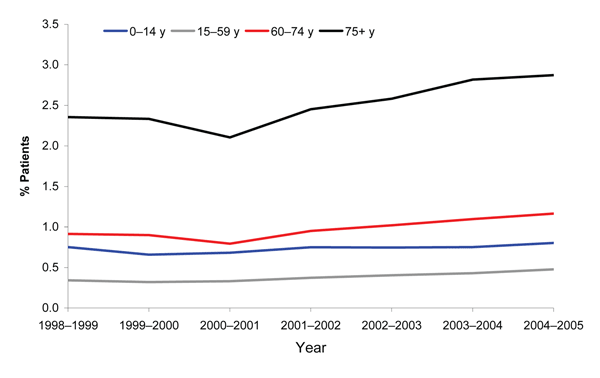

Supplement: Appendix Figure 1 — Percentage of total hospital admissions that were due to pneumonia between 1998–99 and 2004–05, by age group. [file 07-1011_app1-s1.gif]

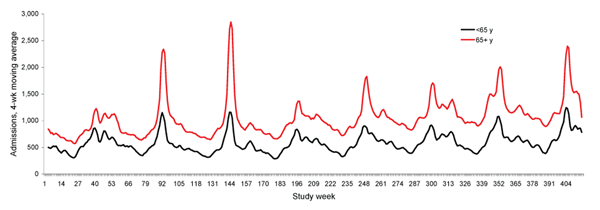

Supplement: Appendix Figure 2 — Seasonal patterns of hospital admissions with a primary diagnosis of pneumonia, by age group (4-week moving average). [file 07-1011_app2-s2.gif]
